# Case report: Fatal insulin overdose in a dog with type 1 diabetes mellitus—characteristics and successful management

**DOI:** 10.3389/fvets.2023.1255701

**Published:** 2023-10-31

**Authors:** Jun-Hyeong Park, Ju-Hyun An, Se-Hoon Kim, Han-Sol Choi, Tae-Hyeon Kim, Ye-In Oh, Kyoung-Won Seo, Hwa-Young Youn

**Affiliations:** ^1^Laboratory of Veterinary Internal Medicine, Department of Veterinary Clinical Science, College of Veterinary Medicine, Seoul National University, Seoul, Republic of Korea; ^2^Department of Veterinary Emergency and Critical Care Medicine and Institute of Veterinary Science, College of Veterinary Medicine, Kangwon National University, Chuncheon-si, Republic of Korea; ^3^Department of Veterinary Internal Medicine, College of Veterinary Medicine, Kyungpook National University, Daegu, Republic of Korea

**Keywords:** dog, glucagon, hypoglycemia, NPH, mannitol

## Abstract

Administering more than 10 times the therapeutic dose of insulin is extremely rare in diabetic dogs and is life threatening with hypoglycemia and seizures if not accompanied by appropriate treatment. A 15-year-old, castrated male miniature poodle dog managed for diabetes presented with depression, disorientation, ataxia, and cluster seizures. The dog had been administered 11.1 U/kg of neutral protamine hegadorn (NPH) insulin (10 times the prescribed dose) 3 h before the onset of symptoms. Blood analysis revealed hypoglycemia, with a circulating glucose level of <50 mg/dL. To treat the hypoglycemia-induced seizures, dextrose was repeatedly administered intravenously. Repeated generalized seizures were treated with anticonvulsants and intermittent mannitol. Since refractory hypoglycemia persisted 24 h after the insulin overdose, it was decided to proceed with glucagon treatment (15–30 ng/kg/min titrated to the blood glucose level after a loading dose of 50 ng/kg intravenous bolus infusion). After 37 h of glucagon treatment, blood glucose levels stabilized. After entering a hyperglycemic state, NPH insulin was administered to manage insulin-dependent diabetes mellitus. This is the first case documented of successful treatment with glucagon, anticonvulsants and intermittent mannitol for refractory hypoglycemia and seizure caused by fatal insulin overdose. Thus, it has great clinical value in veterinary medicine.

## 1. Introduction

Type 1 diabetes mellitus, also known as insulin-dependent diabetes mellitus, develops when the beta cells responsible for producing insulin are severely or almost entirely depleted. Among dogs, this form of diabetes is the most prevalent, and it necessitates insulin injections for the regulation of blood sugar levels, as the name implies. The adverse effects of insulin are attributed to improper use and use of inappropriate doses (1–3), with hypoglycemia being one of the most common adverse effects.

Hypoglycemia is defined as BG concentration < 3.3 mmol/L [60 mg/dL] (4). Clinical manifestations and severity depend on the degree of hypoglycemia, rate of BG decline, and duration of hypoglycemia. Clinical signs of hypoglycemia, such as altered mentation, weakness, ataxia, and seizures, have been reported (5). Although hypoglycemia is one of the most common significant treatment-limiting side effects in diabetic patients, reports on treating hypoglycemia due to insulin overdose in veterinary medicine are rare. In human medicine, current guidelines and quality responsibility indicators for insulin treatment focus almost exclusively on preventing hyperglycemia (5–7).

Management of insulin overdose poses a clinical challenge and may require modified treatment strategies, given the lack of consensus and guidelines in veterinary medicine. Therefore, we present a case of refractory hypoglycemia due to a massive overdose of intermediate-acting NPH insulin in a canine patient with DM and discuss an integrative approach for managing this case.

## 2. Case presentation

A 15-year-old castrated male miniature poodle (body weight 5.37 kg) presented to the emergency room with clinical signs of disorientation, ataxia, and cluster seizures. According to the patient’s medical history, the dog had been managed for DM with NPH insulin application after suffering from diabetic ketoacidosis with acute pancreatitis 5 years earlier. Two years prior, the patient was started on oral hypoglycemic agent to improve BG control. The patient’s home medication was subcutaneous NPH insulin (6 U/dose twice a day), oral hypoglycemic agent (DWP16001, 0.025 mg/kg once every 3 days), and a prescription diet (Royal Canin diabetic) (8, 9). The patient was confirmed to have been administered 60 U of NPH insulin (10 times the prescribed dose) 3 h before the onset of symptoms. As a result of the history check, it was confirmed that the owner accidentally injected too much insulin. The patient’s vital signs were as follows: body temperature 38.0°C; heart rate 134 b/min; respiratory rate 30 b/min; and systolic blood pressure 151 mmHg. No remarkable findings were confirmed on auscultation. Blood analysis revealed hypoglycemia with a circulating glucose level < 2.8 mmol/L (50 mg/dL), and other metabolic causes of generalized seizures, such as electrolyte imbalances, hyperammonemia, and uremia, were ruled out. Hypoglycemia was considered the cause of the seizures, and dextrose 0.5 g/kg intravenous (IV) injection and oral dextrose feeding were started immediately.

**Figure 1:**
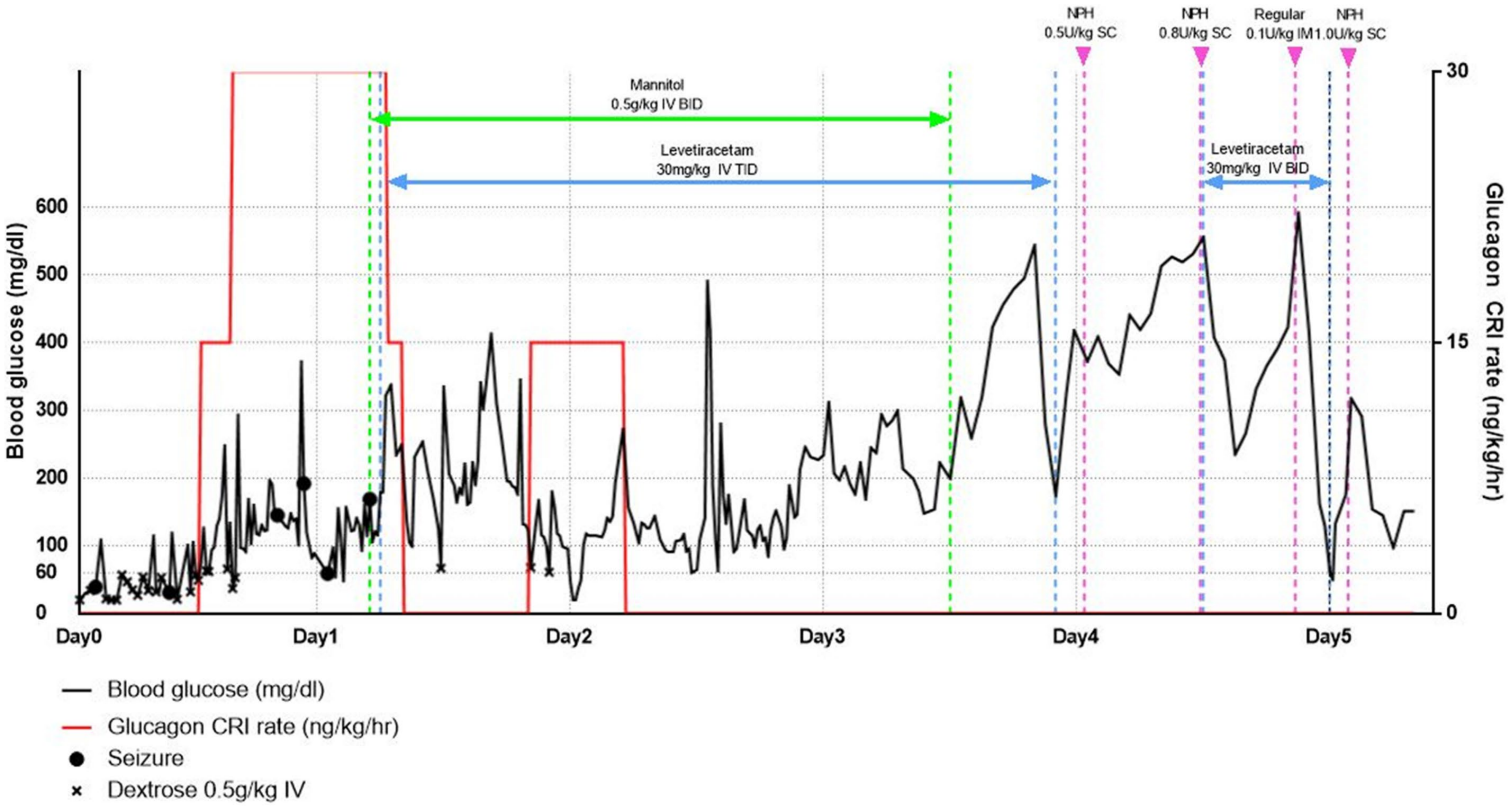
Blood glucose levels over time from first presentation (line graph) and since supply of dextrose, glucagon, anticonvulsants, and mannitol.

In addition, continuous dextrose infusions were administered with 5% dextrose water and 5% dextrose saline at 2.5 mL/kg/h with periodic BG assessments using continuous glucose monitoring (CGM, Freestyle Libre Sensor) during hospitalization. Additionally, whenever BG was <3.3 mmol/L (60 mg/dL), dextrose (0.5 g/kg) was injected IV bolus until the BG rose to ≥5.0 mmol/L (90 mg/dL; Figure 1). Despite the supply of glucose for 1 h, seizures continued to recur; therefore, midazolam (0.2 mg/kg IV) was injected and repeated according to the seizure status. Levetiracetam (30 mg/kg, IV, three times daily) was administered. In addition, mannitol (0.5 g/kg IV for 30 min, twice a day) was administered to reduce swelling of the brain secondary to the seizures. Despite repeated glucose supply, refractory hypoglycemia persisted 24 h after insulin overdose, so it was decided to proceed with glucagon treatment (at a rate of 15 to 30 ng/kg/min flexibly depending on the BG status after a loading dose of 50 ng/kg IV bolus infusion) (5, 10, 11). Glucagon CRI was discontinued if the BG level remained >11.1 mmol/L (200 mg/dL) for more than 1 h; if the BG level decreased to 5.0 mmol/L (90 mg/dL), it was restarted at a rate of 15 ng/kg/min to stabilize the BG level. Glucagon treatment was stopped 37 h after the start of glucagon administration. There were no seizures for 72 h, and the mannitol treatment was discontinued.

At 96 h after admission, the BG level increased to >27.8 mmol/L (500 mg/dL); Although refractory hypoglycemia was improved, severe hyperglycemia was confirmed. At this time, no remarkable findings were identified as a result of the physical and neurological examination. Accordingly, NPH insulin therapy was initiated to improve hyperglycemia and manage insulin-dependent diabetes. First, 0.5 U/kg of NPH insulin was administered and evaluated with a nadir of 19.4 mmol/L (350 mg/dL) and a duration of 0 h. Next, the insulin dose was gradually increased by 10–20% of the previous dose, depending on the patient’s fasting BG level, lowest BG level, and duration. If a rapid rise in BG was confirmed during hospitalization, an additional 0.1 U/kg of regular insulin was administered. After 130 h of hospitalization, the patient returned home with a prescription for NPH insulin, 1 U/kg, twice a day (Figure 1). Nine days later, the patient revisited the hospital; the patient’s vitality and appetite gradually improved, and there were no specific clinical symptoms. Subsequently, the DM was managed with insulin alone in accordance with T1DM; it has been confirmed that the patient is doing well without additional clinical symptoms, including neurological symptoms.

## 3. Discussion

This case reports the use of a glucagon CRI and anti-epileptic medication to manage massive insulin overdose-induced refractory hypoglycemia and seizures in a dog with DM.

The patient, in this case, was diagnosed with insulin-dependent diabetes mellitus 5 years ago after being hospitalized for DKA with pancreatitis. And this patient received a dose of intermediate-acting insulin, NPH, of 11.1 U/kg, which is 10 times the dose normally administered for this patient. The duration of action of dog insulin NPH is 4 to 10 h, and in one study involving well-controlled diabetic dogs, the average dose of NPH insulin was 0.63 U/kg (range 0.4–0.97) injected every 12 h (12). However, in the case of this report, for BG stabilization, dextrose was supplied for 24 h, but stabilization failed, and BG was stabilized 37 h after additional glucagon was supplied. As a result, the time it took for hypoglycemia to improve after excessive insulin injection was more than 96 h, and severe hyperglycemia was confirmed afterwards.

In cases of insulin overdose, the duration of action may be affected by several factors, including the route and site of administration, injection volume, solution concentration, local blood supply, and lipodystrophy or cutaneous amyloidosis at the injection site (13). For example, insulin injections of larger volumes are absorbed more slowly from the subcutaneous space than smaller volumes, which may result in delayed hypoglycemia following the injection. Other factors, such as the presence of insulin autoantibodies and renal dysfunction, can also prolong the duration of hypoglycemia (13–17). Therefore, it may be difficult to predict the duration of hypoglycemia in cases of insulin overdose (18, 19). Based on reported cases involving insulin overdose, reports in veterinary medicine are limited; however, in humans, hypoglycemia has been reported up to several days after injection.

The initial treatment for hypoglycemia should primarily include oral carbohydrate intake (4, 20). However, parenteral glucose should be administered if the patient cannot ingest carbohydrates or hypoglycemia persists. Treatment of hypoglycemic patients generally requires glucagon CRI treatment if dextrose supplementation does not stabilize BG (5, 11).

Glucagon is a 29-amino acid polypeptide hormone secreted by pancreatic α-cells. Its primary function is to maintain glucose production in the liver via glycogenolysis and gluconeogenesis principally in the liver (21). While exogenous glucagon finds utility in various clinical scenarios, including its application in Type 1 Diabetes Mellitus (T1DM) and its approval for emergency management of hypoglycemia, it is worth noting that there are only two documented canine cases involving insulinoma with concurrent hypoadrenocorticism (5) and refractory hypoglycemia resulting from a renal nephroblastoma (22). Furthermore, research on this topic within the field of veterinary medicine, specifically in small animals, remains limited. In addition, there are currently no reports in the canine literature describing continuous intravenous glucagon infusion as a stand-alone treatment or as an adjunct to intravenous dextrose for the management of life-threatening hypoglycemia following an insulin overdose in T1DM.

In this patient’s case, since BG was not stabilized by the glucose supply alone, glucagon CRI treatment was performed, and it was confirmed that BG was stabilized without adverse effects. Based on this case, although many patients suffering from insulin overdose-induced hypoglycemia respond well to glucose supplementation, the addition of glucagon can be considered for patients with persistent hypoglycemia despite standard therapy.

Previous studies have shown that severe hypoglycemia causes brain damage in the cortex and hippocampus and that the degree of injury is closely related to the presence of seizure-like activity (23, 24). Additionally, the osmotic gradient associated with glucose tends to exacerbate any brain edema present, enhancing brain damage (25). Although the mechanism underlying this effect is not yet clear, mannitol added to the infusion to equilibrate the osmolality of the hypoglycemic and hyperglycemic solutions osmotically reduces the resulting brain edema and prevents brain damage (24). In this patient, hypoglycemia occurred due to an insulin overdose, and seizure symptoms developed. To improve this condition, an anticonvulsant and mannitol were administered together. As the BG level was well managed, the seizure symptoms also improved, and it has been 6 months since the anticonvulsant was discontinued, and the patient was managed without additional seizures.

Some limitations should be considered in this case study. In this particular patient’s case, hyperglycemia was confirmed at the 72 h without any episodes of hypoglycemia for the preceding 12 h. However, Insulin treatment was not initiated at this juncture but rather at the 96 h. It is pertinent to acknowledge that both human and veterinary medicine currently lack standardized guidelines for the optimal timing of insulin re-administration following an insulin overdose. A previous study has reported instances of delayed recurrence of hypoglycemia in cases involving insulin overdoses, suggesting that extended observation periods may be necessary (15, 26). Consequently, in this specific case, the decision to administer insulin at the 96 h was made after thorough monitoring, even though hyperglycemia had been confirmed without any associated hypoglycemia at 72 h. But, due to the delayed administration of insulin, although this patient did not exhibit clinical symptoms related to hyperglycemia, but rather experienced severe hyperglycemia. Persistent hyperglycemia, even after the resolution of hypoglycemia, carries the risk of causing neurological damage (27). Thus, timely insulin intervention to alleviate hyperglycemia is imperative. Delayed administration of insulin in such circumstances may lead to adverse effects associated with hyperglycemia. Further reports and research endeavors are warranted to establish guidelines and recommendations regarding the appropriate timing of insulin re-administration following the resolution of hypoglycemia resulting from an insulin overdose.

In conclusion, insulin overdose often requires intensive care admission and frequent monitoring of BG levels, electrolytes, and clinical signs, such as seizures. To the best of our knowledge, this is the first report of the successful and safe use of intravenous glucagon in managing refractory hypoglycemia following an insulin overdose in a dog with T1DM. In addition, despite return to normal BG levels, convulsions continued to occur, and in this regard, seizure symptoms were effectively managed with mannitol treatment to reduce intracranial pressure as well as anticonvulsants. In cases of insulin overdose, hypoglycemia, and hypoglycemic seizures managed with dextrose, glucagon, anti-epilepsy drugs, mannitol, and continuous monitoring can lead to good outcomes.

## Data availability statement

The original contributions presented in the study are included in the article/Supplementary material, further inquiries can be directed to the corresponding author.

## Ethics statement

Written informed consent was obtained from the participant/patient for the publication of this case report.

## Author contributions

J-HP: Conceptualization, Investigation, Writing – original draft. J-HA: Conceptualization, Investigation, Writing – original draft. S-HK: Investigation, Methodology, Writing – review & editing. H-SC: Investigation, Methodology, Writing – review & editing. T-HK: Investigation, Methodology, Writing – review & editing. Y-IO: Investigation, Supervision, Writing – review & editing. K-WS: Investigation, Supervision, Writing – review & editing. H-YY: Investigation, Supervision, Writing – review & editing.
